# Effectiveness and safety of tocilizumab for COVID-19: a systematic review and meta-analysis of randomized clinical trials

**DOI:** 10.1590/1516-3180.2022.0170.R1.01072022

**Published:** 2022-09-12

**Authors:** Paula Ribeiro Lopes Almeida, Osmar Clayton Person, Maria Eduarda dos Santos Puga, Maria Fernanda Giusti, Ana Carolina Pereira Nunes Pinto, Aline Pereira Rocha, Álvaro Nagib Atallah

**Affiliations:** I MD. Otorhinolaryngologist and Postgraduate Student in Evidence-Based Health, Universidade Federal de São Paulo (UNIFESP), São Paulo (SP), Brasil; II MD, PhD. Full Professor, Department of Otorhinolaryngology, Universidade Santo Amaro (UNISA), São Paulo (SP), Brazil; III MD, PhD. Librarian, Information specialist at Cochrane Center in Brazil, São Paulo (SP), Brazil; Director, Library Network, Universidade Federal de São Paulo (UNIFESP), São Paulo (SP), Brazil.; IV Audiologist of Rhinomed, Hospital Brasil, Rede D’OR, Santo André (SP), Brazil.; V PhD. Physiotherapist and Professor, Evidence-Based Health Program, Universidade Federal de São Paulo (UNIFESP), São Paulo (SP), Brazil; Professor, Department of Biological and Health Sciences, Universidade Federal do Amapá (UNIFAP), Macapá (AP), Brazil; Volunteer Researcher, Cochrane Brazil, São Paulo (SP), Brazil.; VI MSc. Pharmacist and Doctoral Student, Evidence-Based Health Program, Universidade Federal de São Paulo (UNIFESP), São Paulo (SP), Brazil; Volunteer Researcher, Cochrane Brazil, São Paulo (SP), Brazil.; VII MD, PhD. Full Professor and Head of the Discipline of Emergency Medicine and Evidence-Based Medicine, Universidade Federal de São Paulo (UNIFESP), São Paulo (SP);; Director of Cochrane Brazil, São Paulo (SP), Brazil.

**Keywords:** COVID-19, SARS-CoV-2, Tocilizumab [supplementary concept], COVID-19 treatment, Respiratory failure, COVID-19 pneumonia

## Abstract

**BACKGROUND:**

Tocilizumab is an anti-human interleukin 6 receptor monoclonal antibody that has been used to treat coronavirus disease 2019 (COVID-19). However, there is no consensus on its efficacy for the treatment of COVID-19.

**OBJECTIVE:**

To evaluate the effectiveness and safety of tocilizumab for treating COVID-19.

**DESIGN AND SETTING:**

Systematic Review of randomized controlled trials (RCTs), Universidade Federal de São Paulo (UNIFESP), São Paulo (SP), Brazil.

**METHODS:**

We searched MEDLINE via PubMed, EMBASE, CENTRAL, and IBECS for RCTs published up to March 2021. Two authors selected studies and assessed the risk of bias and the certainty of the evidence following Cochrane Recommendations.

**RESULTS:**

Eight RCTs with 6,139 participants were included. We were not able to find differences between using tocilizumab compared to standard care on mortality in hospitalized patients with COVID-19 (risk ratio (RR) 0.97, 95% confidence interval (CI) 0.84 to 1.13; 8 trials; 5,950 participants; low-certainty evidence). However, hospitalized patients under tocilizumab plus standard care treatment seemed to present a significantly lower risk of needing mechanical ventilation (risk ratio = 0.78; 95% CI 0.64−0.94 moderate-certainty of evidence).

**CONCLUSIONS:**

To date, the best evidence available shows no difference between using tocilizumab plus standard care compared to standard care alone for reducing mortality in patients with COVID-19. However, as a finding with a practical implication, the use of tocilizumab in association to standard care probably reduces the risk of progressing to mechanical ventilation in those patients.

**REGISTRATION:**

osf.io/qe4fs.

## INTRODUCTION

### Description of the condition

Over 160 million cases of coronavirus disease 2019 (COVID-19) have been reported around the world, with more than 3.3 million deaths.^1^ The COVID-19 pandemic, initiated in 2020, encouraged extraordinary efforts on research regarding pharmacological interventions and vaccines. Despite that, few pharmacological interventions have shown to be effective in the treatment of COVID-19.

COVID-19 infection is similar to Middle East respiratory syndrome and severe acute respiratory syndrome (SARS-COV-1),^2^ with two phases of development: the intense viral replication followed by the immune system response, flooding the host with proinflammatory cytokines. The uncontrolled inflammatory response leads to severe acute respiratory syndrome, which represents the worst prognostic factor in patients with COVID-19.^3^ Interleukin-6 (IL-6) is released as part of the acute-phase response. When higher levels are achieved, the probability of severe coronavirus disease and risk of mechanical ventilation are elevated.^4,5,6^

### Description of the intervention

Tocilizumab (TCZ) is an anti-human IL-6 receptor monoclonal antibody that inhibits IL-6 signaling by blinding soluble and membrane IL-6 receptors. The drug has long been used for rheumatoid arthritis, juvenile inflammatory arthritis, and refractory giant cell arteritis.^7^

### How the intervention might work

COVID-19 creates a hyperinflammatory condition, activated by a cytokine cascade. Of all cytokines identified so far, IL-6 is most closely connected to disease severity.^7^ TCZ inhibits IL-6 action and might be a way to reduce COVID-19 severe cases.

### Why it is important to do this review

Several observational studies have been conducted on treating COVID-19 and they suggest that TCZ is beneficial for moderate, severe, or critical cases of COVID-19.^8,9,10^ However, non-randomized studies may report spurious associations mainly arising from the introduction of confounding factors into the comparative groups, and relying on such results may lead to the introduction of potentially hazardous interventions into clinical practice. Randomized clinical trials (RCTs) became available only by the end of 2020 and they have, so far, shown mixed results for mortality. Therefore, systematic reviews evaluating the effects of tocilizumab considering only RCTs are urgently needed.

## OBJECTIVES

The aim of this review was to evaluate the effectiveness and safety of tocilizumab for treating COVID-19.

## METHODS

### Criteria for considering studies for this review

We undertook a systematic review including only RCTs. Participants must have been diagnosed with COVID-19 by one of the following methods: real time reverse-transcriptase polymerase chain reaction, serum immunoglobulin M antibody assay, or clinical evaluation (typical computed tomographic scan with signs of pneumonia). We included trials evaluating the effect of tocilizumab used alone or in combination with standard care or other interventions.

### Outcomes

Our primary outcome was mortality. Secondary outcomes included the need for mechanical ventilation, days until discharge from hospital, and adverse events.

### Search methods for identification of studies

The search was for all relevant published and unpublished trials without restrictions on language, year, or publication status. Electronic search included PubMed (1966-2021), EMBASE (1974-2021), CENTRAL – 2021 (Cochrane Library) and BVS portal. All RCTs published up to 03/24/2021 were considered for inclusion. Search strategies for each database are provided in Appendix 1. References of included trials were checked to identify additional, relevant trials. When necessary, authors were contacted.

### Study selection and data extraction

All abstracts and reports identified by the search were retrieved and independently evaluated by two authors. If the reference appeared relevant to the review topic, the full text was obtained. The same two authors assessed and selected any relevant trials according to the review's eligibility criteria. In the presence of any disagreements, a third author was consulted.

### Assessment of risk of bias and certainty of evidence

The risk of bias in each trial was assessed by two independent authors. We assessed the methodological quality of each included study using the risk of bias (RoB 2.0) tool as per the Cochrane recommendations. We evaluated the following domains: risk of bias arising from the randomization process, risk of bias due to deviations from the intended interventions (effect of assignment to intervention), missing outcome data, risk of bias in measurement of the outcome, risk of bias in selection of the reported result, and overall risk of bias. Each study was evaluated on all six domains and for each domain the evaluations were scored by assigning the classifications “low risk of bias”, “some concerns of risk of bias”, or “high risk of bias”.^11^ We used the GRADE (Grading of Recommendations Assessment, Development and Evaluations) approach to classify the strength of evidence as very low, low, moderate, or high.^12^ We evaluated the following criteria: risk of bias, inconsistency, imprecision, and indirectness. We summarized the findings, considering the primary outcomes from comparisons, using the GRADE pro platform.

### Measures of treatment effect

We estimated the effects of tocilizumab treatments for our predefined outcomes. Relative risks with their 95% confidence intervals (CI) were estimated using Review Manager 5.4.1 software (London, United Kingdom). We pooled data from the included studies using the generic inverse variance method with a random-effects model. We assessed heterogeneity using the I2 statistic.^13^ The interpretation of I2 depends on the magnitude and direction of the effect as well as the strength of evidence for heterogeneity. We used the following thresholds to assess I2: 0% to 40%: likely not important; 30% to 60%: moderate heterogeneity; 50% to 90%: substantial heterogeneity; 75% to 100%: considerable heterogeneity.

## RESULTS

### Results of the search

**Figure 1.**
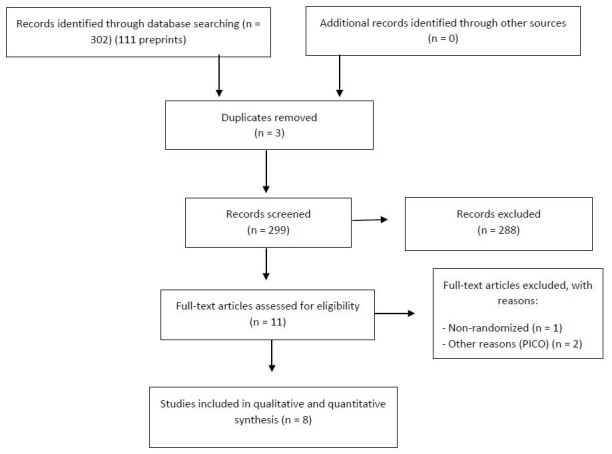
Study flow diagram.

Our database search strategies yielded 413 records. After excluding duplicated reports and reports that were clearly irrelevant or not directly related to the review question, we assessed eleven full-text studies for further scrutiny. Eight multi-center RCTs^7,14–20^ with 6,139 participants were finally included in our systematic review (Figure 1). Details of each trial are described in Table 1.

PICO = Population, Intervention, Comparison, and Outcome.

**Table 1.** Characteristics of the included studies

Study/Country	Participants	Interventions	Outcome
REMAP-CAP Investigators et al.^17^/United Kingdom	350 adults’ participants (age 61.4) hospitalized with moderate, severe, or critical pneumonia (O_2_ &gt; 3 L/minutes, WHO Clinical Progression Scale [WHO-CPS] score ≥ 5 due to COVID-19	Standard care (glucocorticoids) plus single dose TCZ (8 mg/kg − up to 800 mg) versus Standard Care alone	All-cause mortality Time point: 21 days
Hermine et al.^7^/France	131 adults’ patients (age 64.0) hospitalized with moderate-to-severe COVID-19 pneumonia	Standard care (no information) plus single dose TCZ (8 mg/kg − up to 800 mg) versus Standard Care alone	All-cause mortality Need of mechanical ventilation Time point: 4 and 14 days
Tone et al.^14^/United States	243 adults’ patients (age 59.8) with severe acute respiratory syndrome coronavirus 2 (SARS-CoV-2) infection, hyperinflammatory states, and at least two of the following signs: fever (body temperature &gt; 38 °C), pulmonary infiltrates, or the need for supplemental oxygen in order to maintain an oxygen saturation greater than 92%	Standard Care (no information) plus single dose TCZ (8 mg/kg − up to 800 mg) versus- Standard care alone	All-cause mortality Need of mechanical ventilation Time point: 28 days
Salama et al.^18^/United States	389 adults’ participants (age 55.9) hospitalized with COVID-19 with blood oxygen saturation below 94% while breathing ambient air	Standard care (antivirals; glucocorticoids - methylprednisolone, supportive care) plus one or two doses of TCZ (8 mg/kg − up to 800 mg) versus Standard Care plus placebo	All-cause mortality Need of mechanical ventilation Time point: 28 and 60 days
Veiga et al.^15^/Brazil	129 adults’ participants (age 60) with confirmed covid-19 who were receiving supplemental oxygen or mechanical ventilation and had abnormal levels of at least two serum biomarkers (C reactive protein, D dimer, lactate dehydrogenase, or ferritin)	Standard Care (no information) plus single dose TCZ (8 mg/kg − up to 800 mg) versus Standard care alone	All-cause mortality Need of mechanical ventilation Time point: 14 and 30 days
Rosas et al.^19^/United States	438 adult participants (age 60.9) hospitalized with Severe COVID-19	Standard Care (antivirals; low-dose glucocorticoids, convalescent plasma) plus single dose TCZ (8mg/kg − up to 800 mg versus Standard care alone	All-cause mortality Need of mechanical ventilation Time point: 28 and 60 days
RECOVERY Collaborative Group^16^/ United Kingdom	Patients hospitalized (age 63.3) with COVID-19 with hypoxia (oxygen saturation &lt; 92% on air or requiring oxygen therapy) and evidence of systemic inflammation (C-reactive protein [CRP] ≥ 75 mg/L)	Standard care (no information) plus single dose TCZ (8 mg/kg − up to 800 mg) versus Standard Care alone	Need of mechanical ventilation Time point: 28 and 180 days
Salvarani et al.^22^/Italy	123 adult participants (age 60) hospitalized with COVID-19 Pneumonia, with a partial pressure of arterial oxygen to fraction of inspired oxygen (PaO2/FIO2) ratio between 200 and 300 mm/Hg, an inflammatory phenotype defined by a temperature greater than 38 °C during the last 2 days, and/or serum CRP levels of 10 mg/dL or greater and/or CRP level increased to at least twice	Standard Care (no information) plus single dose TCZ (8 mg/kg − up to 800 mg) versus Standard care alone	Need of mechanical ventilation Time point:14 days

### Characteristics of included studies

Participants over 18 years from Europe and South and North America were randomized in each included trial into two groups: standard care alone or associated with tocilizumab 8 mg/kg (maximum dose of 800 mg/day). Tocilizumab was administered to participants as soon as they were randomized.

Standard care was not specified in the majority of the trials. All trials used tocilizumab (8 mg/kg) as soon as the participants were randomized. A second dose was given in most trials if the participant did not improve their clinical status within 24 hours after the first dose. BAAC^14^ and TOCIBRAS^15^ used only one dose. Important baseline characteristics of the participants and interventions are described in Table 1. All trials included hospitalized patients with moderate to severe COVID-19.

### Risk of bias in included studies

**Figure 2.**
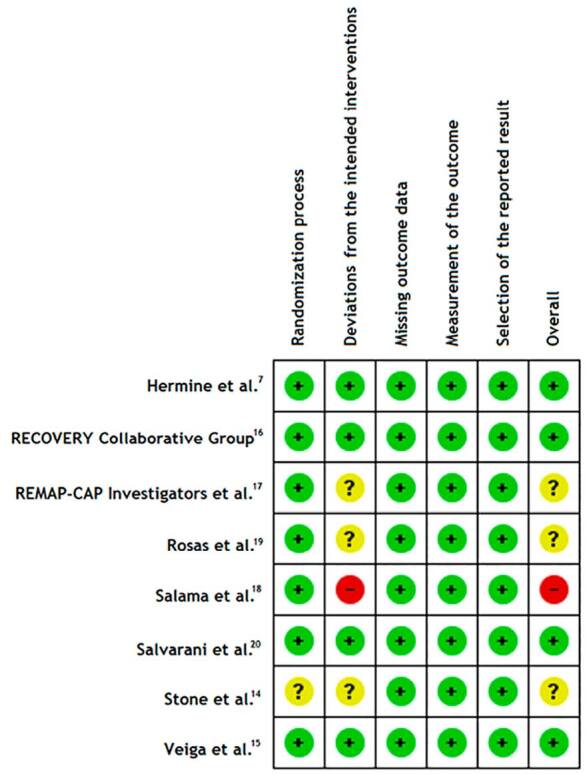
Risk of bias.

The RCTs were assessed by RoB 2.0 (Figure 2). Three of them were judged as being of some concern regarding the risk of bias, four of them were judged as having low risk of bias and only one was graded as having high risk of bias. The most penalized domain was deviation from intended interventions, which occurred mainly because of lack of blinding and/or inappropriate analyses (intention-to-treat).

### Effect of intervention

#### Certainty of evidence

We rated the certainty of the evidence using the GRADE approach. We found low certainty of evidence for the all-cause mortality outcome (Table 2). For that outcome, we downgraded one level due to methodological limitation (risk of bias) and one level due to imprecision (the 95% CI included both a benefit and harm, showing imprecision of the estimated effect). We found moderate certainty of evidence for need of mechanical ventilation. For that outcome, we downgraded one level due to methodological limitation (risk of bias).

**Table 2.** GRADE analysis.^24^

Certainty assessment							Summary of findings				
Participants (studies) Follow-up	Risk of bias	Inconsistency	Indirectness	Imprecision	Publication bias	Overall certainty of evidence	Study event rates (%)		Relative effect(95% CI)	Anticipated absolute effects	
							With standard care	With Tocilizumab		Risk with standard care	Risk difference with Tocilizumab
**All-cause mortality**											
5,950(8 RCTs)	serious^a^	not serious	not serious	serious^b^	none	⊕⊕○○Low	761/2686 (28.3%)	810/3264 (24.8%)	**RR 0.97** (0.84 to 1.13)	28 per 100	**1 fewer per 100** (from 5 fewer to 4 more)
**Need of mechanical ventilation**											
4,705(6 RCTs)	serious^c^	not serious	not serious	not serious	none	⊕⊕⊕○Moderate	365/2230 (16.4%)	329/2475 (13.3%)	**RR 0.78** (0.64 to 0.94)	16 per 100	**4 fewer per 100** (from 6 fewer to 1 fewer)

CI = confidence interval; RR = risk ratio; RCTs = randomized clinical trials.

**Explanations**

a. We downgraded one level because three studies (n = 1,075) had some concerns on the risk of bias and one study (n = 377) had a high risk of bias.

b. We downgraded one level because the 95% CI includes both no effect and a possible benefit.

c. We downgraded one level because two studies (n = 515) had some concerns on the risk of bias and one study (n = 377) had a high risk of bias.

#### All-cause mortality

**Figure 3.**
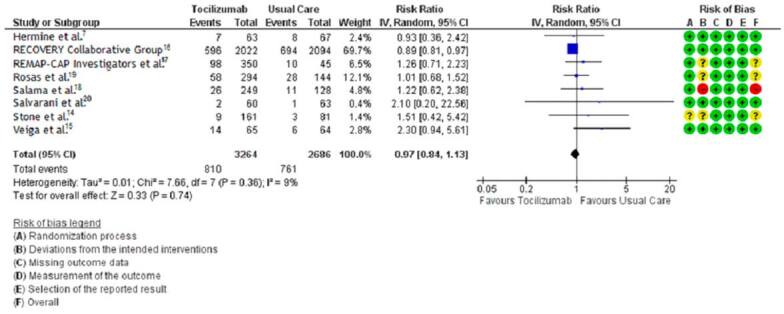
Mortality in COVID-19 patients under tocilizumab plus standard care vs. standard care alone

We were not able to find any difference in mortality of patients with COVID-19 between tocilizumab plus standard care compared to standard care alone (risk ratio [RR] 0.97, 95% CI 0.84 to 1.13; 8 trials; 5,950 participants; low-certainty evidence) (Figure 3).

### Need for mechanical ventilation

**Figure 4.**
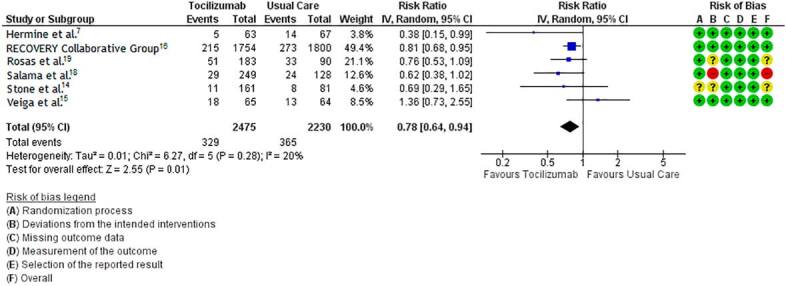
Need of mechanical ventilation in COVID-19 patients under tocilizumab plus standard care versus standard care alone

Patients with COVID-19 treated with tocilizumab plus standard care presented significantly lower risk of progressing to mechanical ventilation when compared to those receiving standard care alone (RR 0.78, 95% CI 0.64 to 0.94; 6 trials; 4,705 participants; moderate certainty of evidence) (**Figure 4**).

A few trials, including EMPACTA,^18^ COVACTA,^19^ TOCIBRAS,^15^ and RECOVERY^16^, described the number of days from the beginning of the trial to participants’ discharge. The average number of days to discharge in the Tocilizumab group was 13.5 days (standard deviation [SD] = 7.5) and in the standard care group was 17.9 (SD = 11.6).

Adverse events were reported in all trials. No difference was found between groups in any trial. Minor events (non-fatal) included variations on hepatic enzymes, neutropenia, thrombosis, hypersensitivity, and anemia.

## DISCUSSION

In this systematic review, including only RCTs assessing the effects of tocilizumab in patients with COVID-19, we found moderate-certainty evidence from six RCTs demonstrating that the use of tocilizumab in combination with standard care was effective for the reduction of need for mechanical ventilation in hospitalized patients with COVID-19. Additionally, we were not able to find any difference between using tocilizumab in association with standard care or standard care alone on mortality in hospitalized patients with COVID-19.

A previous review found no positive effect of using tocilizumab in COVID-19. However, this review included non-randomized trials.^21^ Of note, non-randomized trials may have confounding factors in the comparative groups which often leads to spurious associations.^22^ Relying on such results may lead to the introduction of potentially hazardous interventions into clinical practice.

Tocilizumab, a drug capable of controlling massive inflammation caused by IL-6, has begun to be studied globally. Many observational studies were completed up to the end of 2020, when the first randomized trials were published. These trials were important because the first studies could not come to a conclusion on tocilizumab effectiveness.

Effects on mortality were not observed in participants receiving tocilizumab. It is possible that this intervention is not capable of dealing with the inflammatory discharge of the disease that includes multiple types of interleukins and tumor necrosis factors.^23^ Another possible explanation is that the elevation of interleukins is only part of the normal body reaction to the infection, and its suppression achieves no benefit. Finally, it is possible that the presence of a highly heterogeneous comparison group, using different pharmacological treatments, notably the concomitant administration of corticosteroid therapy, could have influenced our final results for this outcome. Further RCTs should report cointerventions and should minimize bias by stratification of those patients at randomization. As a consequence, a balanced use of comedications could be guaranteed after randomization in future analyses.

It should also be emphasized that most of the included studies recruited moderate to severe COVID-19 patients. Therefore, these results should not be generalized to mild COVID-19 patients. Furthermore, even among patients with moderate to severe ­COVID-19, more trials are needed to determine the best dosage and timing for initiating tocilizumab. Of note, we did not find a significant effect of tocilizumab on the risk of adverse events. Although no safety concerns associated with tocilizumab were observed in our analysis, it should be noted that the best dosage and timing for initiating tocilizumab still need to be further investigated. All included studies used the tocilizumab standard dose: 8 mg per kilogram of body weight (one or two doses, up to 800 mg). Another problem that we saw was the heterogeneity of the basic treatment in the comparative groups. There were variations in medications and doses that did not allow us to rule out interference in the final results found for the treatment.

Some observational studies of tocilizumab treatment have described reduction in the need for invasive mechanical ventilation, or death. Many trials claimed that using tocilizumab in early stages may alter the results. In our subgroup analysis this evidence was not confirmed. Time from beginning of the disease ends just when the inflammatory stage begins and the latter is the bigger problem.

All included studies had limitations related to blinding and treatment allocation. This is another concern of ours and, combined with the degree of moderate certainty that we found, suggests the need for new RCTs.

We suggest carrying out new quality RCTs, with a balanced use of comedications in both groups, so that the question can be answered more robustly. These studies should be standardized as to the basic parameters for describing clinical trial results, such as using the CONSORT Statement (Consolidated Standards of Reporting Trials).

## CONCLUSIONS

The best evidence available showed no difference between tocilizumab plus standard care compared to standard care alone for reducing mortality in patients with COVID-19. However, as a further result with a practical implication, the use of tocilizumab in association to standard care seemed to reduce the risk of progressing to mechanical ventilation in those patients. There is a need for further high-quality randomized double-blind studies using rigorous methodology.

## Appendix 1.

**Table:** Search strategy

**MEDLINE via PubMed** #1 “COVID-19” [Supplementary Concept] OR (COVID 19) OR (COVID-19) OR (2019-nCoV) OR (nCoV) OR (Covid19) OR (SARS-CoV) OR (SARSCov2 or ncov*) OR (SARSCov2) OR (2019 coronavirus*) OR (2019 corona virus*) OR (Coronavirus (COVID-19)) OR (2019 novel coronavirus disease) OR (COVID-19 pandemic) OR (COVID-19 virus infection) OR (coronavirus disease-19) OR (2019 novel coronavirus infection) OR (2019-nCoV infection) OR (coronavirus disease 2019) OR (2019-nCoV disease) OR (COVID-19 virus disease) #2 “tocilizumab” [Supplementary Concept] OR (RHPM-1) OR (RG-1569) OR (R-1569) OR (MSB11456) OR (MSB-11456) OR (atlizumab) OR (monoclonal antibody, MRA) OR (RO-4877533) OR (Actemra) OR (Roactemra) #3 #1 AND #2 = 62 Filters applied:⮽Clinical Trial, Meta-Analysis, Randomized Controlled Trial, Systematic Review.⮽Clear ⮽ all
**COCHRANE LIBRARY** #1 (COVID-19) OR (COVID 19) OR (COVID-19) OR (2019-nCoV) OR (nCoV) OR (Covid19) OR (SARS-CoV) OR (SARSCov2 or ncov*) OR (SARSCov2) OR (2019 coronavirus*) OR (2019 corona virus*) OR (Coronavirus (COVID-19)) OR (2019 novel coronavirus disease) OR (COVID-19 pandemic) OR (COVID-19 virus infection) OR (coronavirus disease-19) OR (2019 novel coronavirus infection) OR (2019-nCoV infection) OR (coronavirus disease 2019) OR (2019-nCoV disease) OR (COVID-19 virus disease) #2 tocilizumab OR (RHPM-1) OR (RG-1569) OR (R-1569) OR (MSB11456) OR (MSB-11456) OR (atlizumab) OR (monoclonal antibody, MRA) OR (RO-4877533) OR (Actemra) OR (Roactemra) #3 #1 AND #2 = 144
**EMBASE** #1 ‘covid 19’/exp OR (COVID 19) OR (COVID-19) OR (2019-nCoV) OR (nCoV) OR (Covid19) OR (SARS-CoV) OR (SARSCov2 or ncov*) OR (SARSCov2) OR (2019 coronavirus*) OR (2019 corona virus*) OR (Coronavirus (COVID-19)) OR (2019 novel coronavirus disease) OR (COVID-19 pandemic) OR (COVID-19 virus infection) OR (coronavirus disease-19) OR (2019 novel coronavirus infection) OR (2019-nCoV infection) OR (coronavirus disease 2019) OR (2019-nCoV disease) OR (COVID-19 virus disease) #2 ‘tocilizumab’/exp OR Actemra OR (actemra 200) OR atlizumab OR lusinex OR (r 1569) OR (r1569) OR roactemra #3 #1 AND #2 = 86 #1 AND #2 AND ([cochrane review]/lim OR [systematic review]/lim OR [meta analysis]/lim OR [controlled clinical trial]/lim OR [randomized controlled trial]/lim) #4 AND [embase]/lim NOT ([embase]/lim AND [medline]/lim)
**BVS PORTAL** #1 MH:”Infecções por Coronavirus”;OR (Infecções por Coronavirus) OR (Infecciones por Coronavirus) OR (Coronavirus Infections) OR (COVID-19) OR (COVID 19) OR (Doença pelo Novo Coronavírus (2019-nCoV)) OR (Doença por Coronavírus 2019-nCoV) OR (Doença por Novo Coronavírus (2019-nCoV)) OR (Epidemia de Pneumonia por Coronavirus de Wuhan) OR (Epidemia de Pneumonia por Coronavírus de Wuhan) OR (Epidemia de Pneumonia por Coronavírus de Wuhan de 2019-2020) OR (Epidemia de Pneumonia por Coronavírus em Wuhan) OR (Epidemia de Pneumonia por Coronavírus em Wuhan de 2019-2020) OR (Epidemia de Pneumonia por Novo Coronavírus de 2019-2020) OR (Epidemia pelo Coronavírus de Wuhan) OR (Epidemia pelo Coronavírus em Wuhan) OR (Epidemia pelo Novo Coronavírus (2019-nCoV)) OR (Epidemia pelo Novo Coronavírus 2019) OR (Epidemia por 2019-nCoV) OR (Epidemia por Coronavírus de Wuhan) OR (Epidemia por Coronavírus em Wuhan) OR (Epidemia por Novo Coronavírus (2019-nCoV)) OR (Epidemia por Novo Coronavírus 2019) OR (Febre de Pneumonia por Coronavírus de Wuhan) OR (Infecção pelo Coronavírus 2019-nCoV) OR (Infecção pelo Coronavírus de Wuhan) OR (Infecção por Coronavirus 2019-nCoV) OR (Infecção por Coronavírus 2019-nCoV) OR (Infecção por Coronavírus de Wuhan) OR (Infecções por Coronavírus) OR (Pneumonia do Mercado de Frutos do Mar de Wuhan) OR (Pneumonia no Mercado de Frutos do Mar de Wuhan) OR (Pneumonia por Coronavírus de Wuhan) OR (Pneumonia por Novo Coronavírus de 2019-2020) OR (Surto de Coronavírus de Wuhan) OR (Surto de Pneumonia da China 2019-2020) OR (Surto de Pneumonia na China 2019-2020) OR (Surto pelo Coronavírus 2019-nCoV) OR (Surto pelo Coronavírus de Wuhan) OR (Surto pelo Coronavírus de Wuhan de 2019-2020) OR (Surto pelo Novo Coronavírus (2019-nCoV)) OR (Surto pelo Novo Coronavírus 2019) OR (Surto por 2019-nCoV) OR (Surto por Coronavírus 2019-nCoV) OR (Surto por Coronavírus de Wuhan) OR (Surto por Coronavírus de Wuhan de 2019-2020) OR (Surto por Novo Coronavírus (2019-nCoV)) OR (Surto por Novo Coronavírus 2019) OR (Síndrome Respiratória do Oriente Médio) OR (Síndrome Respiratória do Oriente Médio (MERS)) OR (Síndrome Respiratória do Oriente Médio (MERS-CoV)) OR (Síndrome Respiratória do Oriente Médio por Coronavírus); OR MH:C01.925.782.600.550.200$ #2 TOCILIZUMAB OR (atlizumab) OR (monoclonal antibody, MRA) OR (RO-4877533) OR (Actemra) OR (Roactemra) #3 #1 AND #2 = 121
